# Axial–Flexural Performance of Steel Fiber-Reinforced Concrete Columns: Effects of Axial Load Ratio and Steel Fiber Volume Fraction

**DOI:** 10.3390/ma19051014

**Published:** 2026-03-06

**Authors:** Sang-Woo Kim, In-Ho Park, Seungwook Seok, Wonchang Choi, Jinsup Kim

**Affiliations:** 1 Department of Civil Engineering, Gyeongsang National University, Jinju 52828, Republic of Korea; 2 Department of Architectural Engineering, Gachon University, Seongnam-si 13120, Republic of Korea

**Keywords:** reinforced concrete column, steel fiber, axial load ratio, monotonic loading, flexural behavior

## Abstract

This study investigates the axial–flexural behavior of steel fiber–reinforced concrete (SFRC) columns under combined constant axial load and monotonic lateral loading. Nine column specimens with different axial load ratios (0.0, 0.10, and 0.20) and steel fiber contents (0.0%, 0.5%, and 1.0%) were tested under monotonic loading to evaluate their failure modes, load–deflection behavior, ductility, and energy absorption capacity. In addition, a sectional P–M interaction analysis was performed to examine the influence of steel fiber inclusion on flexural strength under different axial compression levels. The interaction diagrams indicated that steel fibers expanded the flexural strength envelope, with a more pronounced enhancement in the low-axial-load region. The test results revealed that increasing the axial load ratio enhanced the specimens’ peak load capacity but reduced their ductility, leading to a brittle failure mode. Conversely, the incorporation of steel fiber improved the crack distribution, delayed crack propagation, and enhanced both ductility and energy absorption, particularly under moderate axial load conditions. The failure modes were characterized generally by flexural cracking and localized crushing in the compression zone, with the specimens that contained steel fiber exhibiting a more gradual post-peak load response than the specimens without steel fiber. The energy absorption capacity, quantified as the area under the load–deflection curve, was maximized when the axial load ratio of 0.10 was used in tandem with steel fiber reinforcement, indicating an optimal balance between strength and ductility. Overall, steel fiber inclusion improved deformation capacity and energy absorption under monotonic loading, particularly at low-to-moderate axial load ratios.

## 1. Introduction

As the occurrence of large-scale earthquakes continues to increase worldwide, the need to ensure the reliable performance of reinforced concrete (RC) structures has become increasingly critical [1,2,3,4,5,6,7,8]. Within RC structural systems, columns are essential members that not only support vertical loads but also resist lateral actions, and damage or failure of columns can directly lead to significant degradation of overall structural performance [9,10,11,12,13,14,15,16,17]. Accordingly, there is a growing demand for material and structural alternatives that can enhance the deformation capacity and energy absorption of column members while being implemented through practical and rational detailing [18,19,20,21].

In conventional design practice, densely spaced transverse reinforcement (stirrups) is commonly used to confine the core concrete and secure shear resistance. However, congested transverse reinforcement can cause rebar interference, reduce the quality of reinforcement placement and concrete casting/compaction, and increase construction difficulty and cost. These issues have become more pronounced with the recent surge in steel prices [22,23,24]. Therefore, developing design strategies that can alleviate reinforcement congestion and improve constructability—such as rationally reducing the required amount of transverse reinforcement or allowing wider stirrup spacing while maintaining structural integrity—has emerged as a crucial practical challenge.

From this perspective, steel fiber-reinforced concrete (SFRC) has garnered attention as a promising alternative, as it can enhance the inherent toughness of concrete and control cracking behavior. Steel fibers distributed within the concrete matrix act as bridging elements, effectively restraining crack initiation and propagation, thereby improving tensile strength, flexural toughness, and shear resistance [25,26,27,28,29,30,31]. In other words, the inclusion of steel fibers can mitigate brittle behavior and improve crack distribution, offering the potential to partially reduce reliance on conventional reinforcement detailing.

Previous studies have confirmed the beneficial role of steel fibers primarily in terms of flexural behavior and deformation capacity. Swamy and Al-Ta’an [32] conducted flexural tests on RC beams made with steel fiber-containing concrete and reported that improved post-cracking tensile stress transfer enhanced deformation capacity and ductility while also increasing flexural strength. Lim et al. [33] examined RC beams incorporating steel fibers with different volume fractions and showed that fiber inclusion effectively controlled crack propagation, enhanced ultimate flexural strength, and significantly improved ductility and energy absorption compared to conventional RC beams. These findings suggest that steel fibers can improve the performance of structural members and, under appropriate conditions, may share part of the confinement and crack-control roles, thereby supporting the rationalization of transverse reinforcement detailing.

Nevertheless, the practical use of steel fibers remains largely limited to applications such as substituting girders (SOG) or partially replacing shrinkage reinforcement. To extend SFRC applications to structural members such as columns, further evidence is required regarding how steel fiber inclusion may influence the demand for conventional transverse reinforcement detailing (i.e., stirrup spacing and required reinforcement amount). In particular, it is necessary to quantify how column behavior changes when steel fibers are added, while maintaining the same level of conventional shear reinforcement, and whether increasing the steel fiber volume fraction can be translated into a potential reduction in transverse reinforcement or wider stirrup spacing. Addressing these issues is essential for establishing design guidelines that optimize the role-sharing between steel fiber reinforcement and conventional steel reinforcement, thereby improving constructability while satisfying the required structural performance.

The axial load ratio is also recognized as a key parameter governing column behavior [9,10,11,12,13,14,15,16,17]. Although increasing the axial load ratio generally enhances column flexural strength, it can markedly reduce deformation capacity and increase the risk of compression-dominated failure beyond a certain level. Therefore, it is necessary to examine how the effectiveness of steel-fiber crack bridging and the associated improvement in deformation capacity vary with axial load level, and how this interaction affects the feasibility of rational transverse reinforcement detailing (e.g., wider stirrup spacing). However, many previous studies have investigated either axial load ratio or steel fiber content in isolation, and systematic investigations addressing their combined influence on column behavior remain limited.

To address this research gap, the present study evaluated the axial–flexural behavior of RC columns by considering both the axial load ratio and steel fiber volume fraction as the primary variables. Nine specimens were fabricated by combining three axial load ratios (0.0, 0.10, and 0.20) and three volume fractions (0.0%, 0.5%, and 1.0%) of hooked-end steel fibers, and they were tested under constant axial compression with monotonic lateral loading. Through a comprehensive assessment of failure modes, load–deflection responses, stiffness, strain distribution, ductility, and energy absorption capacity, the interaction between axial load and steel fiber inclusion was clarified. In addition, a P–M interaction analysis was conducted to evaluate the flexural strength enhancement due to steel fiber inclusion under various compressive force levels by comparing the experimental results with conventional flexural strength prediction equations. Ultimately, this study aims to provide fundamental data for developing design strategies in which the inclusion of steel fibers, through improved crack control and deformation capacity, enables a more rational specification of transverse reinforcement detailing.

## 2. Experimental Program

### 2.1. Specimen Design and Fabrication

**Figure 1:**
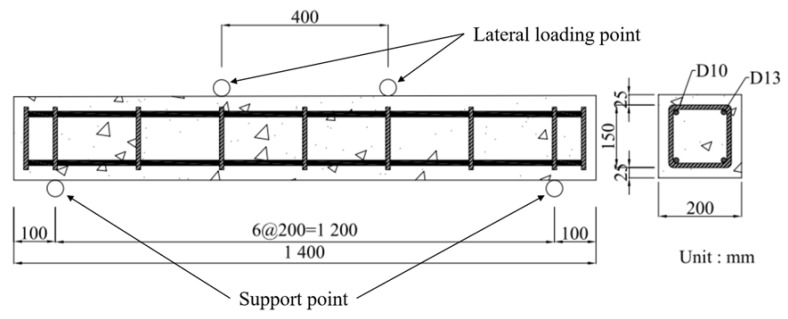
Geometric details of reinforced concrete column test specimen.

Nine specimens were fabricated to investigate the effects of the axial load ratio and steel fiber volume fraction on the performance of RC columns. Figure 1 presents a schematic diagram of a representative specimen and Table 1 provides a summary of the specimen IDs and parameters. The height of each specimen was 1400 mm, with a cross-section of 200 × 200 mm^2^. The concrete cover thickness was 25 mm for all specimens. The longitudinal reinforcement consisted of four deformed reinforcement bars each with a diameter of 13 mm, whereas the transverse reinforcement consisted of nine stirrups each with a diameter of 10 mm. The spacing of the stirrups was 200 mm, with closer spacing (100 mm) provided in the end regions to enhance confinement. To apply the lateral load, loading points were installed 400 mm above and below the column centerline to create a constant bending moment region in the mid-span during the tests.

**Figure 2:**
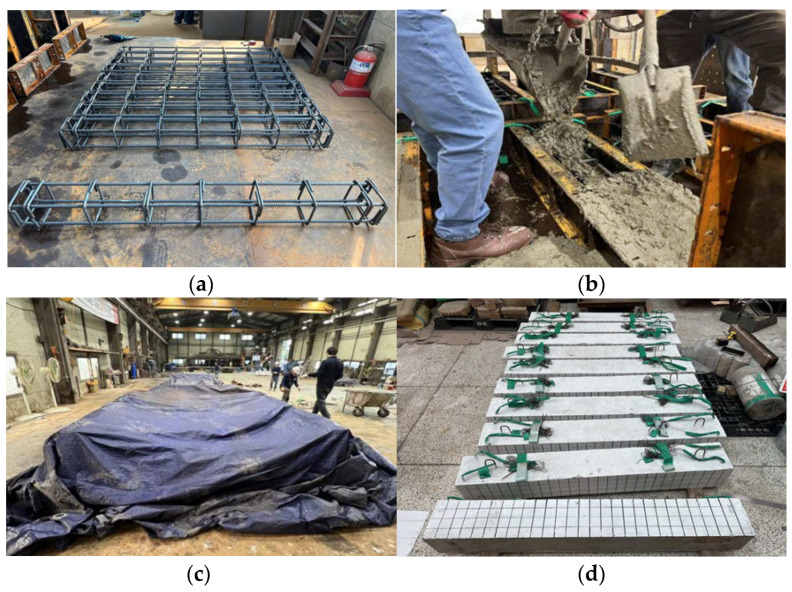
Fabrication process of reinforced concrete column test specimens: (**a**) Steel frame cages; (**b**) Concrete mix cast into forms; (**c**) Cured specimens; (**d**) Fabricated specimens.

Figure 2a–d present the specimen fabrication process. Figure 2a shows the reinforcement steel cages that were assembled based on the design details. Figure 2b shows the concrete, which had been mixed with a specified volume of steel fiber, being cast into the formwork. Following the casting, the specimens were moisture-cured for 28 days using wet burlap and plastic sheets to prevent moisture loss, as shown in Figure 2c. Upon completion of the curing period, the finished specimens were transferred, shown in Figure 2d, to the laboratory for testing.

### 2.2. Material Properties

The mixtures were proportioned with a design target compressive strength of 24 MPa at 28 days. The measured compressive strength of the produced batches was lower (approximately 21 MPa for the plain mixture). Table 2 provides a summary of the mix proportions for each concrete type. The variables include steel fiber volume fractions of 0%, 0.5%, and 1.0%, which correspond to mixture IDs C24-S0, C24-S5, and C24-S10, respectively. All mixes contained ordinary Portland cement, fly ash, fine aggregate (sand), coarse aggregate, and a superplasticizer. The water-to-cement ratio was kept constant for all specimens.

Table 3 shows the mechanical properties of the hooked-end type of steel fiber used in this study. The fiber had a length of 60 mm, diameter of 0.75 mm, and aspect ratio of 80, with tensile strength of 1100 MPa and density of 7850 kg/m^3^. The longitudinal reinforcement (D13) and transverse reinforcement (D10) were both deformed rebar with a nominal yield strength of 400 MPa.

Table 4 presents a summary of the mechanical properties of the concrete mixtures used in this study. The mixtures were proportioned with a design target compressive strength of 24 MPa at 28 days. However, the measured 28-day compressive strength of the plain concrete without steel fibers (C24-S0) was 21.23 MPa, whereas those of the SFRC mixtures with 0.5% (C24-S0.5) and 1.0% (C24-S1.0) volume fractions were 18.32 MPa and 18.87 MPa, respectively. Steel fibers are primarily effective in improving tensile and flexural behavior through crack bridging and post-cracking resistance. In contrast, their influence on compressive strength is generally limited and may vary depending on mixture design and fabrication conditions. In this study, the lower compressive strength observed in the fiber mixtures (approximately 11–14% compared to C24-S0) may be attributed to changes in workability and consolidation conditions during casting. Despite the reduced compressive strength, a significant improvement was evident in the flexural and splitting tensile strengths. The flexural strength increased from 3.30 MPa for C24-S0 to 6.20 MPa for C24-S0.5 and 9.15 MPa for C24-S1.0, while the splitting tensile strength improved from 3.62 MPa for C24-S0 to 4.00 MPa for C24-S0.5 and 4.66 MPa for C24-S1.0. These results indicate that incorporating steel fibers enhances tensile-related properties, particularly flexural performance, by restricting crack propagation through the bridging effect.

### 2.3. Test Set-Up and Measurements

The loading method and test set-up in this study were designed and configured with reference to Kim et al. [34]. The loading sequence was carried out by first applying the axial load up to the target level, followed by the application of the bending moment. The load was applied under displacement control at a constant rate of 2 mm/min and terminated the test when signs of imminent failure were observed in the specimen.

**Figure 3:**
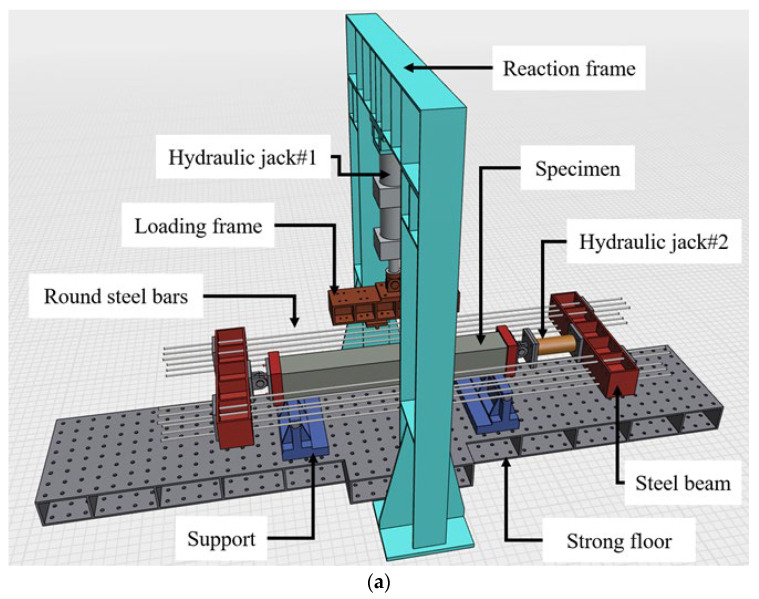

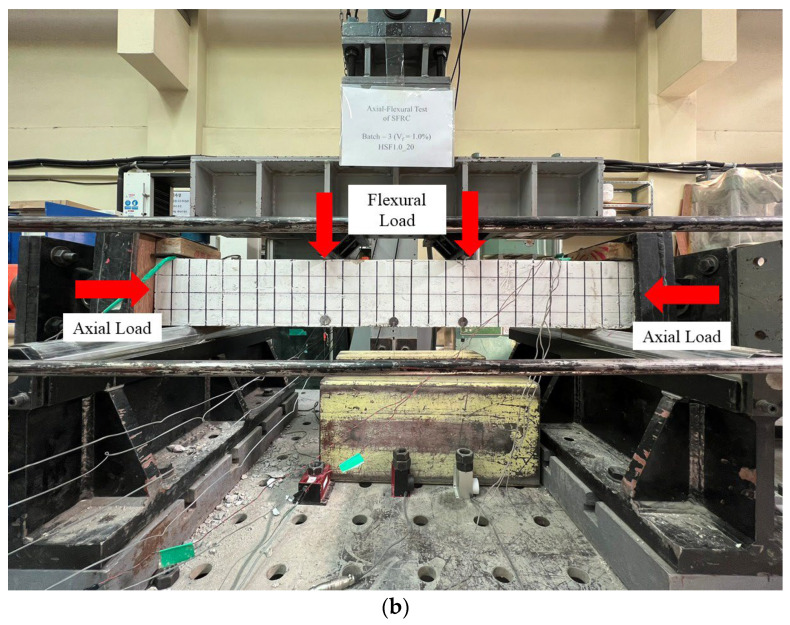
Set-up for testing specimens under compression-bending loads: (**a**) Schematic view of test set-up; (**b**) Photograph of test set-up.

Figure 3a (schematic illustration) and Figure 3b (photo) present the test set-up for the RC column specimens under combined compression–bending loading. As shown, the specimens were horizontally placed on steel supports, with one end connected to the axial loading device and the other end to the lateral loading frame. First, axial load was applied using the Hydraulic Jack #2 set-up, which includes a 1000 kN capacity hydraulic jack, two steel beams, a hinge, twelve round steel bars each with a diameter of 22 mm, steel plates, and high-strength bolts. The steel beams at both ends of the specimen were connected by the round steel bars to enable self-anchoring and to ensure that the applied axial load was uniformly distributed to the specimen. The magnitude of the axial load for each specimen was determined according to the target axial load ratio. In this study, the axial load ratio was defined as 0%, 10%, and 20% of the nominal axial compressive capacity of the column section, P_0_. The value of P_0_ was calculated using the concrete compressive strength from the compression tests and the section/reinforcement properties, as given in Equation (1).

(1) $$P_{0}=0.85\;f_{ck}\;(A_{g}-A_{st})+A_{st}f_{y}$$

where *P*_0_ is the nominal axial compressive capacity of the column section; $f_{ck}$ is the concrete compressive strength (MPa), $f_{y}$ is the yield strength of the longitudinal reinforcement (MPa), $A_{g}$ is the gross cross-sectional area of the specimen (mm^2^), and $A_{st}$ is the total cross-sectional area of the longitudinal reinforcement (mm^2^). The corresponding applied axial load for each specimen is reported in Table 1.

The bending load was applied vertically at two loading points using Hydraulic Jack #1, which created a constant moment region of 400 mm at the mid-span of the column. The distance from the support to the loading point was set to 400 mm, and the total clear span between the supports was 1200 mm. In addition, the strain of the longitudinal reinforcement was measured using strain gauges attached to the rebar. Three linear variable differential transformers (LVDTs) were installed to measure deflection, one at the center of the column and two directly below each loading point, positioned at the lower part of the column. The applied lateral load was measured using a load cell integrated into the actuator and recorded all measurements using a data logger.

## 3. Test Results and Discussion

### 3.1. Failure Mode

**Figure 4:**
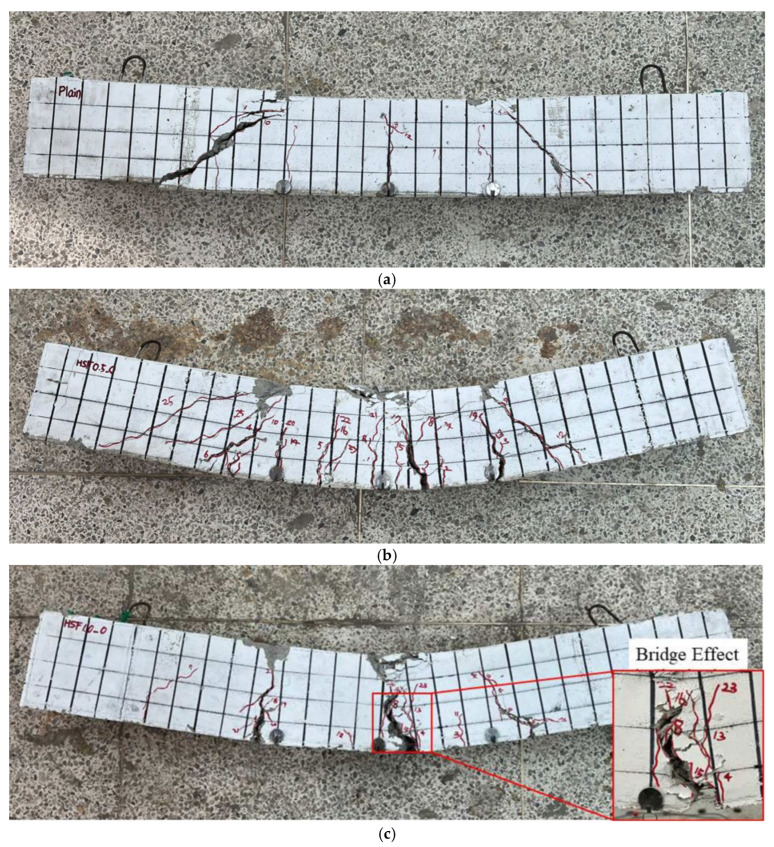

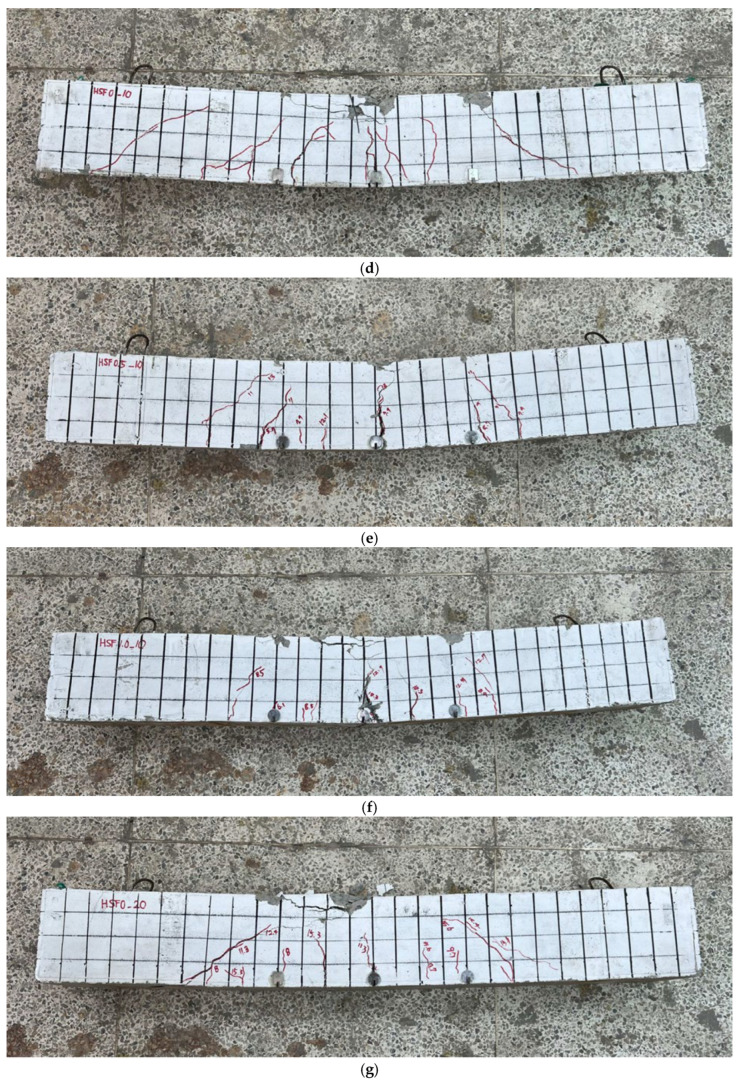

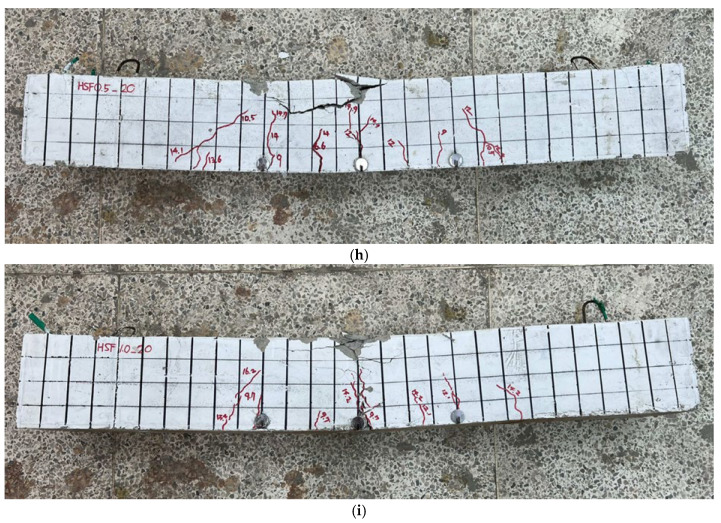
Failure modes of specimens: (**a**) S0-A0; (**b**) S0.5-A0; (**c**) S1.0-A0; (**d**) S0-A10; (**e**) S0.5-A10; (**f**) S1.0-A10; (**g**) S0-A20; (**h**) S0.5-A20; (**i**) S1.0-A20.

Figure 4a–i show the crack patterns and failure modes for all the specimens. Figure 4a shows that the first diagonal crack in S0-A0 occurred at a load of 49.07 kN, followed by the formation of flexural cracks. As the load was increased, the diagonal cracks propagated, and the specimen eventually failed in diagonal (shear) failure mode. Figure 4b shows that S0.5-A0 exhibited its first flexural crack at a load of 49.69 kN. With further loading, flexural cracks developed throughout the specimen, leading to flexural failure. Figure 4c shows that the first flexural crack in S1.0-A0 occurred at 47.24 kN. As the load was increased, cracks at the mid-span propagated, resulting in flexural failure. A bridging effect due to the inclusion of steel fiber is evident at the central cracks. Figure 4d shows that S0-A10 exhibited its first flexural crack at 46.94 kN, followed by the simultaneous development of diagonal cracks and flexural cracks. Additional vertical flexural cracks initiated from the bottom of the member as the load was increased, and the specimen ultimately failed in flexural mode. Figure 4e illustrates that S0.5-A10 experienced its first flexural crack at a load of 47.24 kN. As the load was increased, additional vertical flexural cracks formed at the bottom of the member, and the specimen failed in flexural mode. Compared to S0-A10, the crack propagation range for S0.5-A10 was noticeably reduced. As shown in Figure 4f, S1.0-A10 showed its first vertical flexural crack at the bottom of the member at 56.45 kN. With further loading, the specimen failed in flexural mode. Figure 4g shows that S0-A20 experienced the first flexural crack at a load of 63.41 kN, accompanied by the formation of diagonal cracks similar to those observed in S0-A10. Due to the increased axial load ratio, the failure region in the compression zone expanded, and S0-A20 ultimately failed in flexural mode. Figure 4h indicates that S0.5-A20 exhibited its first flexural crack at 52.14 kN and failed in flexural mode as the load was increased. Similarly to S0-A20, an increase in the failure region of the compression zone is evident due to the higher axial load ratio. Figure 4i shows that, for S1.0-A20, the first flexural crack occurred at a load of 86.34 kN. With an increase in the load, the specimen failed in flexural mode. Although the expansion of the compression zone failure region in S1.0-A20 is not as pronounced as in S0-A20 and S0.5-A20, multiple cracks parallel to the longitudinal axis of the specimen indicate an increase in the compression zone failure area.

Overall, the observed crack patterns indicate that the axial load ratio and steel fiber content govern the transition of dominant damage mechanisms. As the axial load ratio increased (A10 to A20), compressive damage in the concrete compression zone became more pronounced, and the damaged region expanded, suggesting that the response was increasingly controlled by compression-zone crushing. In contrast, the inclusion of steel fibers contributed to crack bridging and improved crack distribution, thereby reducing the extent of crack propagation and delaying crack localization, particularly under low-to-moderate axial load ratios. These observations are consistent with the changes in failure characteristics among the specimen series shown in Figure 4.

### 3.2. Monotonic Load–Deflection Curves and Characteristic Points

**Figure 5:**
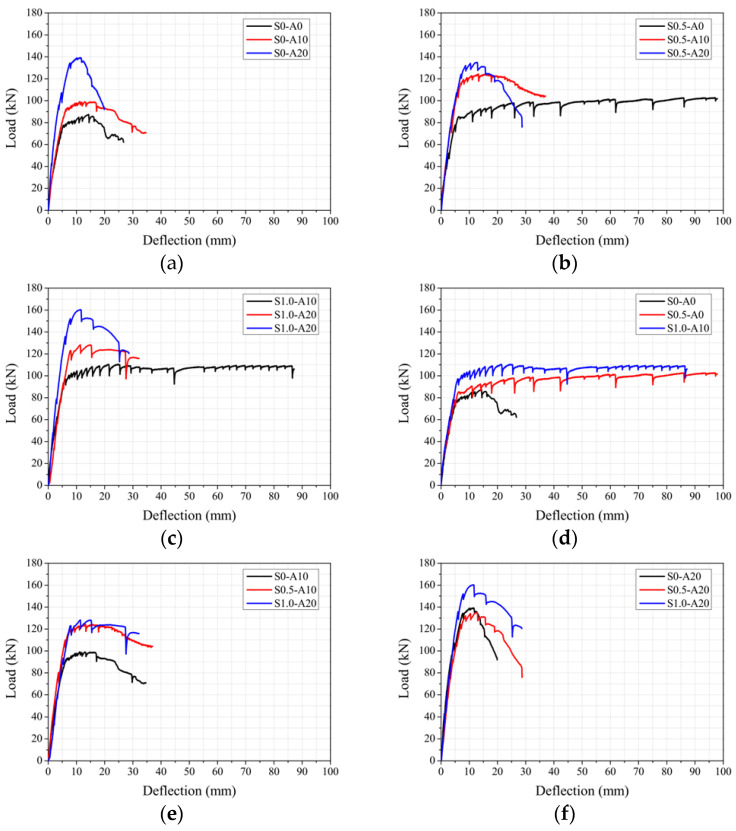
Comparison of monotonic load–deflection response curves of specimens: (**a**) S0 series; (**b**) S0.5 series; (**c**) S1.0 series; (**d**) A0 series; (**e**) A10 series; (**f**) A20 series.

Table 5 presents the mechanical properties at the characteristic points of each specimen as well as the ductility values. These properties include the yield load (Py), yield deflection (∆y), peak load (Pp), peak deflection (∆p), ultimate load (Pu), and ultimate deflection (∆u). The yield load and corresponding deflection were determined at the point where the strain of the longitudinal rebar reached 0.002. The peak point (peak load and corresponding deflection) is defined based on the maximum load during the loading process. In this study, the ultimate point is the load that corresponds to a 10% reduction from the peak load. This was adopted because some specimens did not reach 80% reduction from the peak load before test termination. Figure 5 presents the monotonic load–deflection response curves for the tested specimens.

#### 3.2.1. Effect of Axial Load Ratio

Table 6 provides a summary of the yield and peak behavior of the specimens with different axial load ratios (0.0, 0.10, and 0.20) and different steel fiber volume fractions (0.0%, 0.5%, and 1.0%). The table presents the yield load (Py), yield deflection (∆y), peak load (Pp), and peak deflection (∆p) for each specimen, along with their ratios normalized to the corresponding control specimen with the same steel fiber volume fraction but without axial loading. The results demonstrate that the axial load ratio has a significant influence on both the yield and peak behavior of the specimens. For the S0 series without steel fiber, increasing the axial load ratio from 0.0 (S0-A0) to 0.20 (S0-A20) led to a substantial increase in the yield load from 75.31 kN to 132.69 kN, which corresponds to a ratio of 1.76. Similarly, the peak load increased from 87.44 kN to 139.16 kN (ratio of 1.59). However, the corresponding yield and peak deflections decreased, showing a reduction in the ratio of approximately 0.79, which indicates a diminished deformation capacity at higher axial load ratios. A similar trend is evident for the S0.5 and S1.0 series. In the S0.5 series, the yield load increased by approximately 64% (from 78.35 kN to 128.38 kN), whereas the peak load increased by 38% (from 97.90 kN to 134.95 kN) when the axial load ratio was increased from 0.0 to 0.20. The S1.0 series exhibited a 36% increase in yield load and a 45% increase in peak load over the same axial load range. In all cases, however, both yield and peak deflections decreased as the axial load ratio was increased, with peak deflection ratios dropping to around 0.46 to 0.51. These results indicate that, although higher axial load ratios can improve the load-carrying capacity of the columns, they also can lead to a notable reduction in deformation capacity. This trade-off suggests that an excessively high axial load ratio may increase the likelihood of brittle failure under loading due to the limited ability of the member to undergo large inelastic deformations.

#### 3.2.2. Effect of Steel Fiber Volume Fraction

Table 7 presents a summary of the yield and peak behavior of the specimens with different steel fiber volume fractions (0.0%, 0.5%, and 1.0%) and different axial load ratios (0.0, 0.10, and 0.20). The table presents the yield load (Py), yield deflection (∆y), peak load (Pp), and peak deflection (∆p) for each specimen, along with their ratios normalized to the corresponding control specimen without steel fiber at the same axial load ratio. As the table shows, the increase in steel fiber volume fraction generally had a positive effect on both the yield and peak load behavior of the specimens. For specimens with an axial load ratio of 0.0, increasing the steel fiber volume fraction from 0% to 1.0% resulted in an increase in the yield load from 75.31 kN to 95.65 kN, which corresponds to an approximate 27% improvement. Similarly, the peak load increased from 87.44 kN to 110.54 kN, which indicates an approximate 26% improvement. Moreover, the peak deflection increased from 14.35 mm to 24.69 mm, which indicates a substantial enhancement in deformation capacity. A similar trend is evident for specimens with an axial load ratio of 0.10. The yield load increased by approximately 26% and the peak load increased by about 29% as the steel fiber volume fraction was increased from 0% to 1.0 percent. The peak deflection shows only a slight increase from 11.17 mm to 11.37 mm. In contrast, for specimens with an axial load ratio of 0.20, the effect of steel fiber addition was less pronounced. Although the peak load increased by approximately 15%, the yield load decreased only slightly from 132.69 kN to 129.65 kN. The peak deflection also showed negligible change, from 11.40 mm to 11.29 mm.

The results shown in Table 7 indicate that incorporating steel fiber enhances the tensile strength of the member, restrains crack propagation, and improves ductility. The improvement in deformation capacity due to the inclusion of steel fiber is more significant at lower axial load ratios, where the crack-bridging and toughness-enhancing effects of the fiber could be fully developed. However, at higher axial load ratios, the effect of the steel fiber is limited due to the dominant influence of the compressive force.

#### 3.2.3. Stiffness Analysis

Bending stiffness (K∆) is calculated according to Equation (2). The initial stiffness of each specimen, based on the secant stiffness value, was evaluated at the yield point. Figure 5 presents the stiffness characteristics of the tested specimens under the different axial load ratios and steel fiber volume fractions. Table 8 presents the calculation results.

(2) $$K∆=\frac{P_{Y}-P_{0}}{\Delta_{Y}-\Delta_{0}}$$

As shown in Figure 5 and Table 8, the specimens exhibited clear variations in stiffness values depending on the axial load ratio and steel fiber volume fraction. For the A0 series without axial loading, the initial stiffness of S0-A0, the plain specimen without steel fiber, was 14.85 N/mm. With the steel fiber content of 0.5%, S0.5-A0 showed a decrease in stiffness to 14.59 kN/mm, and with 1.0% steel fiber, S1.0-A0 exhibited an increase to 16.69 kN/mm. These values correspond to approximately −1.8% and +15.1%, respectively, compared with the plain specimen, indicating that steel fiber reinforcement contributes to a stiffer response in the early loading stages. For the A10 series with an axial load ratio of 0.10, the stiffness of the plain specimen was 14.57 kN/mm, whereas the specimens with 0.5% and 1.0% steel fiber reinforcement reached 18.96 kN/mm and 16.14 kN/mm, respectively. These values represent increases of about 23.2% and 9.7%, respectively, compared to the plain specimen, suggesting that the beneficial effect of the inclusion of steel fiber becomes more pronounced under moderate axial load conditions. For the A20 series with an axial load ratio of 0.20, the stiffness of the plain specimen was 16.44 kN/mm, which decreased to 16.09 kN/mm with the inclusion of 0.5% steel fiber and increased to 21.05 kN/mm with 1.0% steel fiber. These values correspond to −2.2% and +21.9%, respectively, relative to the plain specimen. These results confirm that the effectiveness of the inclusion of steel fiber remains significant even under high axial load levels.

Overall, the incorporation of steel fiber reinforcement enhanced the stiffness performance of the specimens under all the axial load conditions, with the effect becoming more evident as the axial load ratio was increased. In particular, S1.0-A20 achieved the highest stiffness value of 21.05 kN/mm, demonstrating the maximum synergistic effect of the combination of axial load and fiber reinforcement.

### 3.3. Strain Analysis

**Figure 6:**
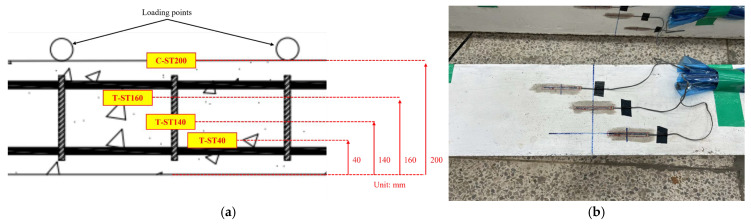
Details of strain-gauge instrumentation for the specimens: (**a**) schematic layout; (**b**) photograph.

**Figure 7:**
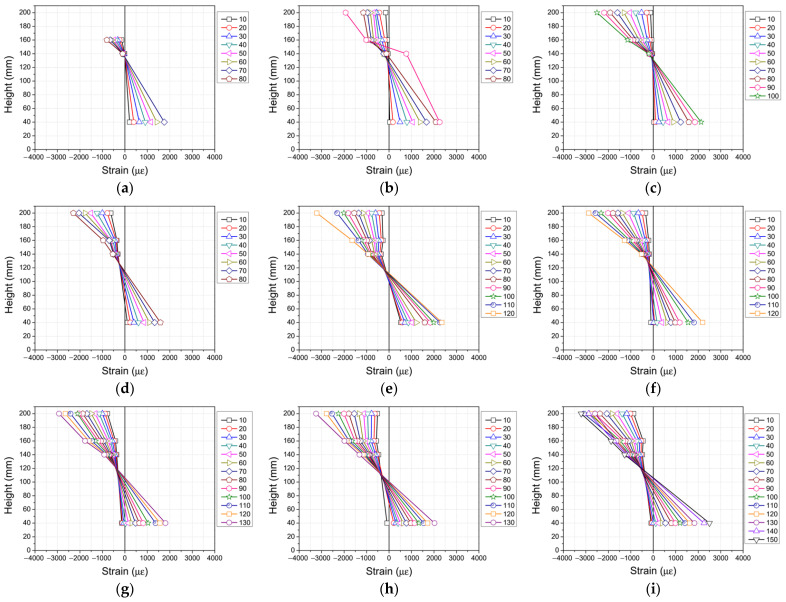
Strain distribution throughout specimens: (**a**) S0-A0; (**b**) S0.5-A0; (**c**) S1.0-A0; (**d**) S0-A10; (**e**) S0.5-A10; (**f**) S1.0-A10; (**g**) S0-A20; (**h**) S0.5-A20; (**i**) S1.0-A20.

The strain distribution along the cross-section at the mid-span section of each specimen was measured to analyze the flexural behavior and deformation characteristics at different loading stages. Strain gauges were attached at a height of 40 mm (T-ST40) on the tensile side and at 140 mm (T-ST140), 160 mm (T-ST160), and 200 mm (C-ST200) on the compressive side as shown in Figure 6. Table 9 presents a summary of the recorded data and Figure 7 shows the strain distributions that correspond to the various loading stages based on the recorded data. For the A0 series (without axial loading), the tensile strain at the bottom of the beam increased sharply as flexural cracks developed and propagated. In contrast, the addition of steel fiber in S0.5-A0 and S1.0-A0 resulted in a more controlled increase in tensile strain. This outcome indicates that the steel fiber provided an effective crack-bridging action that delayed the localization of the strain and promoted a more uniform stress redistribution across the cracked section. For the A10 series, compared to the A0 series, the introduction of axial loading led to higher initial compressive strain and suppressed the development of tensile strain. The specimen without a steel fiber (S0-A10) showed a significant accumulation of strain at higher load levels. However, for the specimens with steel fiber (S0.5-A10 and S1.0-A10), the strain development was more gradual, thus confirming the beneficial effect of steel fiber reinforcement in distributing internal stress and enhancing ductility under combined axial and flexural loading. For the A20 series, the influence of high axial load levels was more pronounced, leading to a substantial increase in compressive strain. The control specimen (S0-A20) exhibited a tendency for strain localization in the compression zone, suggesting the onset of concrete crushing. Conversely, the specimens with steel fiber (S0.5-A20 and S1.0-A20) maintained a more linear strain distribution up to higher load levels, demonstrating both a significant improvement in deformation capacity and the mitigation of premature crushing failure.

Overall, the inclusion of steel fiber contributed to a more uniform strain distribution throughout the specimen column section, regardless of the axial load level. This outcome effectively suppressed localized strain concentrations, thereby mitigating brittle failure in both the tensile and compressive regions. This effect was particularly evident under high axial load conditions (A20 series) where the increased volume fraction of the steel fiber significantly enhanced the deformation capacity of the columns and promoted a more ductile failure model.

### 3.4. Deformation Capacity and Energy Absorption Under Monotonic Loading

#### 3.4.1. Ductility (Deformation Capacity)

Ductility (deformation capacity) and energy absorption are key indices for comparing column performance under lateral loading, and they are evaluated herein based on the monotonic load–deflection response. In this study, ductility (*μ*) was calculated using Equation (3). Table 5 shows the calculation results.

(3) $$\mu =\Delta_{U}\;/\Delta_{Y}$$

**Figure 8:**
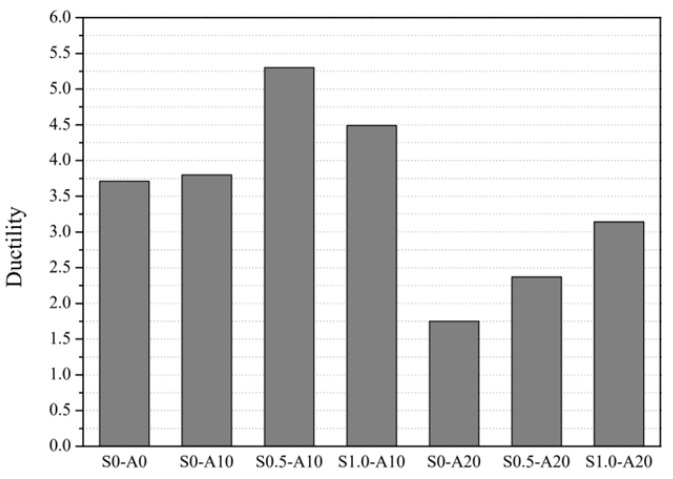
Comparison of ductility values based on axial load ratio and steel fiber volume fraction.

Figure 8 presents the ductility results for the tested specimens. Based on the results shown in both Table 5 and Figure 8, S0.5-A10 exhibits the highest ductility value (μ = 5.30), indicating that the combination of a moderate axial load ratio (0.1) and 0.5% steel fiber provides the most favorable balance between strength and deformation capacity. The resultant improvement can be attributed to the bridging effect of the steel fiber that delays crack propagation and enhances post-yield deformation. S1.0-A10 also demonstrates high ductility (μ = 4.49), confirming the positive contribution of the steel fiber; however, the increase from 0.5% to 1.0% volume fraction resulted in a slight reduction in μ, likely due to a more brittle matrix response at the higher fiber content. In contrast, specimens with the highest axial load ratio (A20) generally showed markedly less ductility. For instance, S0-A20 recorded the lowest μ value (1.75), indicating that greater axial compression suppresses flexural deformation and promotes brittle failure. Even with the inclusion of steel fiber, ductility under high axial load ratios improves only moderately, as observed in S0.5-A20 (μ = 2.37) and S1.0-A20 (μ = 3.14). This trend suggests that the beneficial effect of steel fiber on ductility diminishes as the axial load increases due to the predominant influence of compressive stress on failure behavior. Overall, the results highlight that moderate axial load ratios combined with steel fiber reinforcement significantly enhance ductility, whereas excessive axial loads limit this benefit regardless of fiber content.

#### 3.4.2. Energy Absorption Capacity

**Figure 9:**
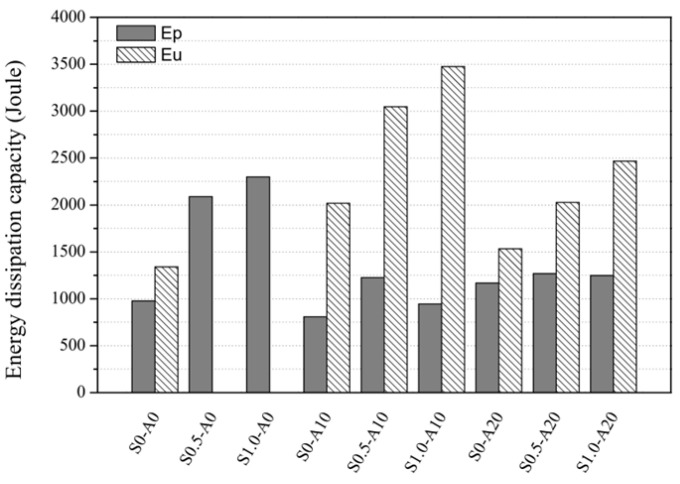
Comparison of energy absorption capacity values.

Figure 9 and Table 10 present the energy absorption capacity results for each specimen, categorized into the area up to the peak load (Ep) and the area up to the displacement that corresponds to 90% of the peak load (Eu). Ep is defined as the area under the load-deflection curve up to the peak load, and Eu is defined as the cumulative dissipated energy up to the point where the load decreases to 90% of the peak load. For the axial load ratio of zero, Ep increased from 978.00 J at a steel fiber volume fraction of 0% to 2088.92 J at 0.5%, representing an increase of approximately 113.6%, and further to 2299.47 J at 1.0%, which represents an approximate 135.1% increase. For the axial load ratio of 0.1, Ep increased from 809.33 J at a steel fiber volume fraction of 0% to 1225.63 J at 0.5%, representing an increase of approximately 51.4%, and to 946.37 J at 1.0%, representing an increase of approximately 16.9%. Eu increased from 2018.93 J at 0% to 3047.08 J at 0.5%, representing an increase of approximately 50.9%, and further to 3476.54 J at 1.0%, which represents an approximate 72.2% increase. For the axial load ratio of 0.2, Ep increased from 1167.57 J at a steel fiber volume fraction of 0% to 1267.73 J at 0.5%, representing an increase of approximately 8.6%, and to 1247.23 J at 1.0%, representing an increase of approximately 6.8%. Eu increased from 1532.74 J at 0% to 2028.91 J at 0.5%, representing an increase of approximately 32.4%, and further to 2468.12 J at 1.0%, which represents an approximate 61.0% increase. Overall, Eu tended to decrease with an increase in the axial load ratio; however, under the same axial load conditions, a higher steel fiber content consistently improved the energy absorption capacity. In conclusion, the incorporation of steel fiber improved both Ep and Eu under all axial load conditions in this study, with the highest energy absorption capacity observed at the axial load ratio of 0.1 for Eu and axial load ratio of zero for Ep.

### 3.5. PM Interaction

In this section, a sectional analysis-based axial load–bending moment (P–M) interaction analysis was conducted to quantitatively examine the effect of steel fiber inclusion under different axial compression levels. The P–M curves were obtained by varying the neutral-axis position, computing the corresponding (P, M) pairs that satisfy force equilibrium, and then determining the associated moment capacity. The tensile contribution of SFRC was incorporated using the model proposed by Kim et al. (2006) [35]. The nominal moment capacity was calculated according to Equation (4).

(4) $$M_{n}=T_{s}\left(d-\frac{a}{2}\right)+T_{f}\left(\frac{D}{2}+\frac{e}{2}-\frac{a}{2}\right)+C_{s}(\frac{a}{2}-d^{\prime })$$

where $T_{s}$ is the tensile force in the longitudinal reinforcement, $T_{f}$ is the tensile force contributed by SFRC, and $C_{s}$ is the compressive force in the compression reinforcement. In addition, $a,\;d,\;d^{\prime },D,\;and\;e\;denote$ the equivalent compression stress-block depth, effective depth, distance to the centroid of compression reinforcement, section depth, and the parameter associated with the SFRC tensile stress block, respectively.

**Figure 10:**
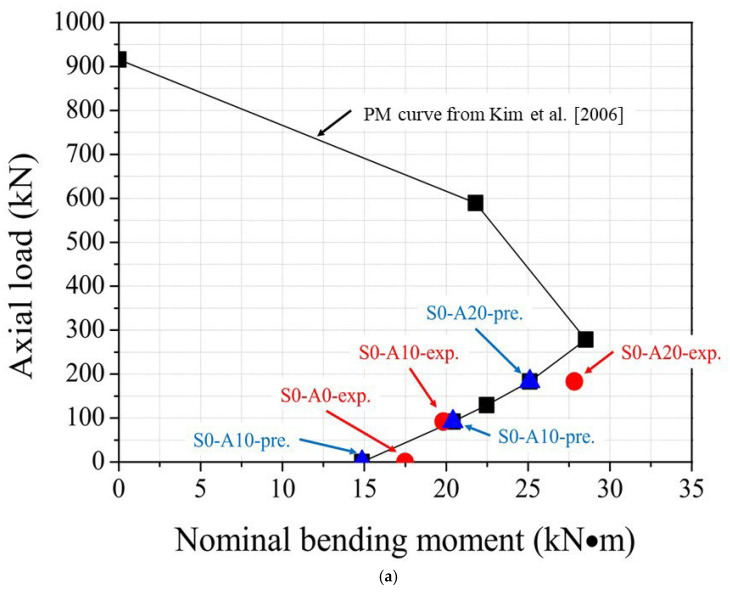

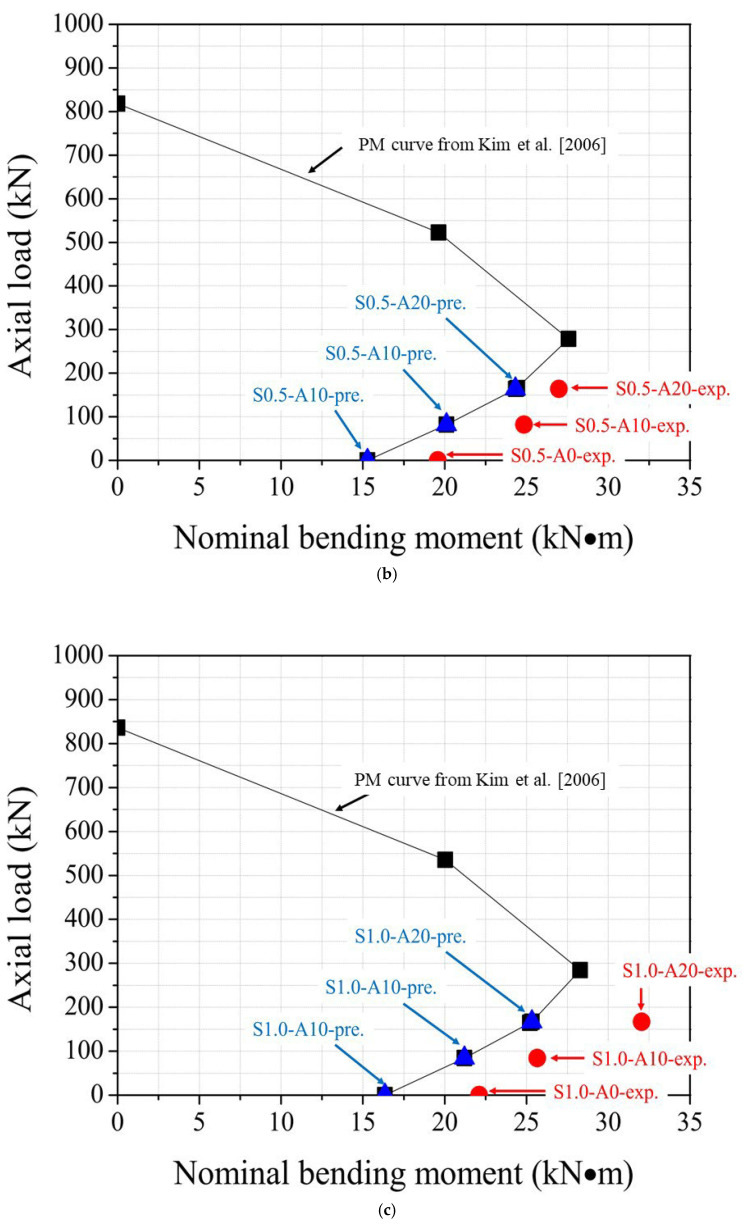
P–M interaction diagrams for RC columns with different steel fiber volume fractions: (**a**) V_f_ = 0.0%, (**b**) V_f_ = 0.5%, (**c**) V_f_ = 1.0% [35].

Table 11 compares the experimental values of the nine specimens with the analytical predictions. Overall, the analysis reproduced the increasing trend in moment capacity with increasing steel fiber volume fraction; however, it generally underestimated the nominal moment for the SFRC specimens. As shown in Figure 10, steel fiber inclusion expanded the P–M interaction envelope, and the enhancement was more pronounced in the low-axial-load (tension-controlled) region, where the tensile resistance provided by fiber bridging is more influential.

The P–M interaction analysis illustrates the potential flexural strength enhancement associated with steel fiber inclusion under various axial load levels and provides a quantitative context for the discussion on rationalizing transverse reinforcement detailing. Nevertheless, because transverse reinforcement detailing was not directly varied in this study, any implication regarding reduced transverse reinforcement demand or wider stirrup spacing should be verified through additional tests with varying confinement details.

## 4. Summary

The test results indicate that the axial load ratio and steel fiber volume fraction govern the dominant damage mechanism and post-peak response of RC columns under constant axial compression and monotonic lateral loading. Increasing the axial load ratio shifted the response toward compression-zone crushing, resulting in a higher peak load but reduced deformation capacity. In contrast, the inclusion of steel fibers improved crack distribution and delayed crack localization through their bridging action. Steel fibers generally enhanced lateral strength and deformation capacity at the same axial load level. For example, at an axial load ratio of 0.10, the peak load increased from 99.08 kN (S0-A10) to 124.26 kN (S0.5-A10) and 128.28 kN (S1.0-A10), while ductility increased to a maximum of μ = 5.30 for S0.5-A10. Energy absorption (area under the monotonic load–deflection curve) increased with steel fiber content, with the largest Eu observed in the A10 series. Finally, the sectional P–M interaction analysis provided a quantitative context for the enhancement of flexural strength due to steel fiber inclusion. The interaction envelopes expanded with increasing fiber volume fraction, particularly in the low-axial-load (tension-controlled) region, supporting the discussion on rationalizing transverse reinforcement detailing.

## 5. Conclusions

This study investigated the axial–flexural behavior of RC columns incorporating steel fibers under constant axial compression and monotonic lateral loading. Nine specimens were tested with axial load ratios of 0.0, 0.10, and 0.20 and steel fiber volume fractions of 0.0%, 0.5%, and 1.0%. The following conclusions are drawn from the experimental and analytical findings:

1. The observed failure patterns indicate a clear transition in the dominant damage mechanism governed by axial load ratio and steel fiber inclusion. As the axial load ratio increased from A10 to A20, compressive damage and localized crushing in the compression zone became more pronounced, leading to a more brittle post-peak response. In contrast, steel fiber inclusion improved crack distribution and delayed crack localization through bridging action, resulting in a more gradual post-peak load reduction, particularly at low-to-moderate axial load ratios.
2. Increasing the axial load ratio enhanced peak load capacity but reduced deformation capacity. For the plain series (S0), the peak load increased from 87.44 kN at A0 to 139.16 kN at A20 (+59%), while ductility decreased from 3.71 to 1.75 (−53%), demonstrating a strength–ductility trade-off associated with increasing compression-zone crushing under higher axial compression.
3. Steel fibers increased peak load and ductility at a given axial load ratio. At A10, the peak load increased from 99.08 kN (S0-A10) to 124.26 kN (S0.5-A10) and 128.28 kN (S1.0-A10), and the maximum ductility was obtained for S0.5-A10 (μ = 5.30). At A20, steel fibers mitigated the ductility loss, with ductility increasing from μ = 1.75 (S0-A20) to 2.37 (S0.5-A20) and 3.14 (S1.0-A20).
4. Energy absorption, defined as the area under the monotonic load–deflection curve, increased consistently with steel fiber content. At A10, Eu increased from 2018.93 J to 3047.08 J (+51%) and 3476.54 J (+72%) as *V*_*f*_ increased from 0.0% to 0.5% and 1.0%. At A20, Eu increased from 1532.74 J to 2028.91 J (+32%) and 2468.12 J (+61%), indicating that steel fibers enhanced energy absorption even under higher axial compression.
5. The sectional P-M interaction analysis provided additional quantitative evidence of flexural strength enhancement due to steel fiber inclusion under varying axial compression levels. The P–M envelopes expanded with increasing steel fiber volume fraction, particularly in the low axial load region. At A10, the experimental nominal moment increased from 19.82 kN·m (S0-A10) to 24.85 kN·m (+25%, S0.5-A10) and 25.66 kN·m (+29%, S1.0-A10), supporting the observed strength improvements and providing analytical context for discussing more rational transverse reinforcement detailing.
6. This study is limited by the use of a single specimen for each parameter set and by the small-scale nature of the specimens. Future work should include repeated tests to establish reproducibility, larger-scale specimens to address size effects, and higher-strength concrete mixtures consistent with current practice to further validate the observed trends and their design implications.

## Figures and Tables

**Table 1:** Reinforced column specimen IDs and parameters.

Specimen ID	Axial Load		Steel Fiber Volume Fraction (%)
	(%)	(kN) *	
S0-A0	0	0	0
S0.5-A0	0	0	0.5
S1.0-A0	0	0	1.0
S0-A10	10	92	0
S0.5-A10	10	82	0.5
S1.0-A10	10	84	1.0
S0-A20	20	183	0
S0.5-A20	20	164	0.5
S1.0-A20	20	167	1.0

* The applied axial load was determined as 0%, 10%, and 20% of the nominal axial compressive capacity calculated by Equation (1).

**Table 2:** Concrete mix proportions.

Mix ID	Water (kg/m^3^)	Cement (kg/m^3^)	Fly Ash (kg/m^3^)	Sand (kg/m^3^)	Coarse Aggregate (kg/m^3^)	Superplasticizer (kg/m^3^)	Steel Fiber (kg/m^3^)	Steel Fiber Volume Fraction (%)
C24-S0	176	328	36	584	917	3.3	0	0.0
C24-S0.5	176	328	36	584	917	3.3	39.3	0.5
C24-S1.0	176	328	36	584	917	3.3	78.5	1.0

**Table 3:** Mechanical properties of steel fiber.

Fiber Type	Length (mm)	Diameter (mm)	Aspect Ratio	Tensile Stress (MPa)	Density (kg/m^3^)
Hooked end	60	0.75	80	1100	7850

**Table 4:** Mechanical properties of concrete.

Mix ID	Steel Fiber Volume Fraction (%)	Compressive Strength (MPa)	Flexural Strength (MPa)	Splitting Tensile Strength (MPa)
C24-S0	0.0	21.23	3.30	3.62
C24-S0.5	0.5	18.32	6.20	4.00
C24-S1.0	1.0	18.87	9.15	4.66

**Table 5:** Characteristic points of specimens.

Specimen ID	Yield Point		Peak Point		Ultimate Point		Ductility **μ**
	Yield Load *P_y_* (kN)	Yield Deflection $\Delta_{y}$ (mm)	Peak Load *P_p_* (kN)	Peak Deflection $\Delta_{p}$ (mm)	Ultimate Load *P_u_* (kN)	Ultimate Deflection $\Delta_{u}$ (mm)	
S0-A0	75.31	5.07	87.44	14.35	78.72	18.79	3.71
S0.5-A0	78.35	5.37	97.90	25.75	-	-	-
S1.0-A0	95.65	5.73	110.54	24.69	-	-	-
S0-A10	91.53	6.28	99.08	11.17	89.08	23.86	3.80
S0.5-A10	101.04	5.33	124.26	13.16	111.82	28.23	5.30
S1.0-A10	115.74	7.17	128.28	11.37	115.74	32.17	4.49
S0-A20	132.69	8.07	139.16	11.40	124.66	14.13	1.75
S0.5-A20	128.38	7.98	134.95	12.86	122.01	18.94	2.37
S1.0-A20	129.65	6.16	160.23	11.29	144.26	19.37	3.14

**Table 6:** Comparison of yield and peak behavior of specimens with different axial load ratios.

Specimen ID	*P_y_* (kN)	Ratio	$\Delta_{y}$ (mm)	Ratio	*P_p_* (kN)	Ratio	$\Delta_{p}$ (mm)	Ratio
S0-A0	75.31	1.00	5.07	1.00	87.44	1.00	14.35	1.00
S0-A10	91.53	1.22	6.28	1.24	99.08	1.13	11.17	0.78
S0-A20	132.69	1.76	8.07	1.59	139.16	1.59	11.40	0.79
S0.5-A0	78.35	1.00	5.37	1.00	97.90	1.00	25.75	1.00
S0.5-A10	101.04	1.29	5.33	0.99	124.26	1.27	13.16	0.51
S0.5-A20	128.38	1.64	7.98	1.49	134.95	1.38	12.86	0.50
S1.0-A0	95.65	1.00	5.73	1.00	110.54	1.00	24.69	1.00
S1.0-A10	115.74	1.21	7.17	1.25	128.28	1.16	11.37	0.46
S1.0-A20	129.65	1.36	6.16	1.08	160.23	1.45	11.29	0.46

**Table 7:** Comparison of yield and peak load behavior of specimens with different steel fiber volume fractions.

Specimen ID	*P_y_* (kN)	Ratio	$\Delta_{y}$ (mm)	Ratio	*P_p_* (kN)	Ratio	$\Delta_{p}$ (mm)	Ratio
S0-A0	75.31	1.00	5.07	1.00	87.44	1.00	14.35	1.00
S0.5-A0	78.35	1.04	5.37	1.06	97.90	1.12	25.75	1.79
S1.0-A0	95.65	1.27	5.73	1.13	110.54	1.26	24.69	1.72
S0-A10	91.53	1.00	6.28	1.00	99.08	1.00	11.17	1.00
S0.5-A10	101.04	1.10	5.33	0.85	124.26	1.25	13.16	1.18
S1.0-A10	115.74	1.26	7.17	1.14	128.28	1.29	11.37	1.02
S0-A20	132.69	1.00	8.07	1.00	139.16	1.00	11.40	1.00
S0.5-A20	128.38	0.97	7.98	0.99	134.95	0.97	12.86	1.13
S1.0-A20	129.65	0.98	6.16	0.76	160.23	1.15	11.29	0.99

**Table 8:** Stiffness values of tested specimens.

Specimen ID	Py (kN)	P0 (kN)	$\Delta_{y}$ (mm)	$\Delta_{0}$ (mm)	$K_{\Delta }$(kN/mm)
S0-A0	75.31	0	5.07	0	14.85
S0.5-A0	78.35		5.37		14.59
S1.0-A0	95.65		5.73		16.69
S0-A10	91.53		6.28		14.57
S0.5-A10	101.04		5.33		18.96
S1.0-A10	115.74		7.17		16.14
S0-A20	132.69		8.07		16.44
S0.5-A20	128.38		7.98		16.09
S1.0-A20	129.65		6.16		21.05

**Table 9:** Strain distribution of specimens.

Specimen ID	Height	Load (kN)														
		10	20	30	40	50	60	70	80	90	100	110	120	130	140	150
S0-A0	160	−141	−234	−318	−408	−494	−577	−655	−802							
	140	−11	−21	−30	−50	−72	−84	−89	−96							
	40	210	397	644	908	1160	1420	1754	(NON)							
S0.5-A0	200	−146	−429	−552	−614	−697	−829	−962	−1142	−1930						
	160	−72.7	−188	−326	−461	−566	−686	−794	−915	−1013						
	140	−42.6	−101	−152	−196	−217	−242	−232	−113	772						
	40	33	175	488	798	1053	1383	1665	2084	2253						
S1.0-A0	200	−120	−289	−523	−781	−1043	−1304	−1592	−1915	−2184						
	160	−68	−154	−266	−384	−504	−628	−749	−886	−1012						
	140	−38	−65	−90	−109	−115	−131	−160	−171	−180						
	40	31	110	247	440	659	899	1221	1587	1859						
S0-A10	200	−622	−781	−999	−1242	−1516	−1775	−2045	−2289							
	160	−354	−387	−442	−527	−633	−653	−694	−964							
	140	−308	−335	−374	−405	−444	−480	−506	−535							
	40	111	242	401	602	850	1092	1326	1584							
S0.5-A10	200	−311	−434	−601	−774	−948	−1152	−1357	−1548	−1816	−2013	−2309	−3213			
	160	−268	−346	−451	−553	−654	−768	−878	−981	−1117	−1222	−1362	−1642			
	140	−345	−413	−484	−542	−595	−648	(NON)	−755	−810	−863	−942	−923			
	40	528	588	704	857	1020	1215	(NON)	1594	1834	1987	2269	2355			
S1.0-A10	200	−339	−494	−660	−874	−1107	−1372	−1576	−1791	−2013	−2320	−2570	−2870			
	160	−205	−276	−352	−444	−542	−652	−735	−825	−920	−1046	−1153	−1277			
	140	−174	−215	−253	−292	−327	−361	−384	−411	−437	−469	−496	−524			
	40	−100	61	19	167	386	620	784	980	1176	1557	1815	2199			
S0-A20	200	−773	−880	−1000	−1140	−1318	−1495	−1691	−1875	−2047	−2104	−2419	−2650	−2931		
	160	−416	−480	−556	−639	−739	−840	−947	−1062	−1179	−1302	−1469	−1605	−1769		
	140	−362	−399	−441	−484	−531	−581	−630	−669	−706	−747	−807	−852	−916		
	40	−142	−107	−47	30	159	306	474	645	819	1037	1366	1561	1807		
S0.5-A20	200	−513	−629	−778	−952	−1126	−1335	−1549	−1786	−2005	−2251	−2534	−2784	−3246		
	160	−563	−673	−794	−917	−1029	−1153	−1275	−1401	−1518	−1643	−1776	−1879	−1986		
	140	−463	−523	−594	−664	−730	−797	−857	−914	−970	−1030	−1098	−1165	−1313		
	40	−92.2	212	306	402	506	630	776	971	1137	1328	1543	1720	2025		
S1.0-A20	200	−869	−1016	−1176	−1380	−1608	−1835	−2065	−2374	−2597	−2841	−3087	−2754	−2591	−2869	−3204
	160	−449	−532	−624	−734	−853	−972	−1092	−1233	−1338	−1460	−1587	−1580	−1636	−1748	−1875
	140	−485	−540	−602	−667	−732	−788	−841	−899	−940	−992	−1048	−1099	−1147	−1201	−1272
	40	−93	−56	12	113	238	391	548	807	988	1192	1411	1618	1835	2261	2502

**Table 10:** Energy absorption capacity of specimens.

Specimen ID	*E_p_* (Joule)	*E_u_* (Joule)
S0-A0	978.00	1339.96
S0.5-A0	2088.92	-
S1.0-A0	2299.47	-
S0-A10	809.33	2018.93
S0.5-A10	1225.63	3047.08
S1.0-A10	946.37	3476.54
S0-A20	1167.57	1532.74
S0.5-A20	1267.73	2028.91
S1.0-A20	1247.23	2468.12

**Table 11:** Comparison between experimental and analytical nominal moment capacities under different axial load levels.

Specimen ID	V_f_	Experimental Value		Predicted Value	
		P-Exp. (kN)	M_n_-Exp. (kN·m)	P-Pre. (kN)	M_n_-Pre. (kN·m)
S0-A0	0	0	17.49	0	14.86
S0-A10		92	19.82	92	20.42
S0-A20		183	27.83	183	25.11
S0.5-A0	0.5	0	19.58	0	15.28
S0.5-A10		82	24.85	82	20.11
S-0.5A20		164	26.99	164	24.32
S1.0-A0	1.0	0	22.11	0	16.35
S1.0-A10		84	25.66	84	21.21
S1.0-A20		167	32.05	167	25.34

## Data Availability

The original contributions presented in this study are included in the article. Further inquiries can be directed to the corresponding authors.
